# Erratum for the Research Article: “Dynamic conservation for migratory species” by M. Reynolds, B. L. Sullivan, E. Hallstein, S. Matsumoto, S. Kelling, M. Merrifield, D. Fink, A. Johnston, W. M. Hochachka, N. E. Bruns, M. E. Reiter, S. Veloz, C. Hickey, N. Elliot, L. Martin, J. W. Fitzpatrick, P. Spraycar, G. H. Golet, C. McColl and S. A. Morrison

**DOI:** 10.1126/sciadv.aat3915

**Published:** 2018-03-30

In the article “Dynamic conservation for migratory species,” Candace Low was previously noted only in the Acknowledgments rather than included in the author list, as was appropriate. The author list and associated affiliations, acknowledgments, and citation sections have been corrected to include her as a full author. The HTML and PDF (full text) have been corrected.

